# Socioeconomic variations in risky sexual behavior among adolescents in 14 sub-Saharan Africa countries who report ever having had sex

**DOI:** 10.1186/s12939-020-01352-8

**Published:** 2021-01-06

**Authors:** Mohamed M. Ali, Leena Merdad, Saverio Bellizzi

**Affiliations:** 1 UNDP-UNFPA-UNICEF-WHO-World Bank Special Programme of Research, Development and Research Training in Human Reproduction (HRP), World Health Organization, 20 Avenue Appia, 1211 Geneva, Switzerland; 2 Faculty of Dentistry, King Abdulaziz University, Jeddah, Saudi Arabia; 3 Partnership for Maternal, Newborn and Child Health, Geneva, Switzerland

## Abstract

**Background:**

Equity is a guiding principle of the Global Strategy for Women, Children and Adolescents’ Health (2016–2030) aimed at improving adolescent health and responding more effectively to adolescents’ needs. We investigated the socioeconomic differentials in having multiple sexual partners and condom use among unmarried adolescents who reported ever having had sex aged 15–19 years in 14 sub-Saharan countries.

**Methods:**

Using the most recent publicly available Demographic and Health Surveys conducted between 2011 and 2018, we calculated survey- and sex-specific proportions of two or more partners and condomless sex, both overall and by selected socioeconomic characteristics and we fitted logistic regression models to estimate the survey- and sex-specific adjusted odds ratios. The pooled adjusted odds ratios were estimated using multilevel logistic regression.

**Results:**

In most countries, higher percentages of male adolescents than female adolescents reported having more than one partner in the last 12 months. Conversely, a lower percentage of young male reported having condomless sex when compared to young female: from 19.8% in Gabon to 84.5% in Sierra Leone among male adolescents and from 32.6% in Gabon to 93.2% in Sierra Leone among female adolescents. In the multilevel analyses, condomless sex was associated with place of residence, wealth and schooling for both female and male adolescents, while among male adolescents multiple partnerships was significantly associated with place of residence.

**Conclusion:**

Our findings on disparities in condomless sex associated with socioeconomic characteristics might reflect constraint choice and decision making. Results also suggest the need for educational programming and services and better access to barrier methods.

**Supplementary Information:**

The online version contains supplementary material available at 10.1186/s12939-020-01352-8.

## Introduction

Adolescents in Africa are at an elevated risk of a number of negative health outcomes associated with early and unsafe sexual activity, including infection with human immunodeficiency virus (HIV) and other sexually transmitted diseases and unintended pregnancy [1]. Although there has been a global decline in risky adolescent sexual behavior [2, 3], this decline has been slow and has not had a large impact on HIV incidence.

Social determinants of health have a profound impact on adolescent health, and evidence supports that underlying structural and proximal determinants affect adolescent health indicators [4]. Gender, as a social determinant, plays a major role in adolescent health [5]. Disparities exist in sexual risk behaviors, as adolescent girls face challenges like forced sexual initiation that compromise their sexual and reproductive health [6]. The Global Strategy for Women, Children and Adolescents’ Health (2016–2030), which was launched in 2015, identifies adolescents as central to achieving the Sustainable Development Goals and provides an unprecedented opportunity to improve adolescent health and to respond more effectively to adolescents’ needs. Equity is one of the guiding principles of the strategy, and the strategy recommends policies that strengthen health care systems to collect, analyze and report health inequality data [7].

Sub-Saharan Africa has the worst profile of adolescent health indicators (such as mortality, reproductive health, behavioral risk factors and noncommunicable diseases) in comparison to that of other regions [8]. Sub-Saharan Africa also carries the largest HIV burden; in 2016, globally approximately 260,000 adolescents between the ages of 15 and 19 years were newly infected with HIV, of whom 200,000 lived in 23 priority countries, which are predominantly sub-Saharan African countries [9]. Young people in the African Region have been reported to engage in risky sexual behaviors such as early sexual debut, engagement in multiple sexual partnerships and condomless sexual intercourse [1, 10, 11]. Many studies have reported associations between these behaviors and social determinants, including place of residence [10, 12], educational status [10, 12], wealth [12, 13], family structure [14] and school attendance [14].

National levels of sexual behavior among adolescents have been documented in some comparative studies; however, very few studies have systematically investigated the roles of place of residence, household wealth and education in influencing risky behaviors in sub-Saharan Africa [15, 16]. These three characteristics are considered proxy measures that partially capture the influence of the community (place of residence), the household affluence level, (household wealth) and knowledge and exposure to information (education), respectively, on adolescent behaviors. Inequality among this age group requires a systematic analysis of the available data to document the experiences of adolescents in different socioeconomic strata to establish a baseline for future monitoring and tracking of changes within these strata and to assess the impact of interventions and policies in reducing socioeconomic inequalities. Therefore, the objective of this paper is to investigate the differentials in risky sexual behavior, namely, having multiple sexual partners and not using condoms at last sexual intercourse among single, never-married adolescent males and females aged 15–19 years who report ever having had sex in 14 sub-Saharan Africa countries. We hypothesized that having multiple partners would be much more common in urban settings because of a greater range of possible partners and exposure to the internet and that condom use would also be more common because of enhanced access. We also hypothesized that the number of sexual partners would be lower among female adolescents in wealthier households because of a lack of need for transactional sex but would be higher for wealthier male adolescents because of their greater ability to attract potential sexual partners. Similarly, we expected condom use to increase with wealth and education because high wealth and education levels enhance individuals’ negotiating abilities and understanding of risk, respectively.

## Methods

We examined the Demographic and Health Surveys (DHS) from sub-Saharan Africa that are on the public domain at the end of July 2020 and retained surveys that were conducted between 2011 and 2018 that have met the inclusion criteria described below. DHS are nationally representative surveys that collect data on numerous population and health topics. DHS surveys collect primary data from households using a household questionnaire and from eligible female respondents using a women’s questionnaire. In some surveys, data is collected from a subsample of men using a men’s questionnaire [17].

The analysis population comprised single (never-married) female and male adolescents aged 15–19 years who reported having had sex. We focused on two self-reported behavioral outcomes: number of sexual partners in the last 12 months (a subset of adolescents who ever had sex) and condom use at most recent sexual intercourse among those who reported having had sex in the last 3 months (a subset of adolescents who had sex in the last 12 months). The 3-month cut off for condom use among sexually active respondents was chosen rather than the more commonly used 12-month cut off because of doubts about the meaning of reported “current” condom use by adolescents who have been sexually inactive for several months. To ensure a sufficient sample size for disaggregated analysis as well as model stability and convergence, we retained surveys with at least 200 female and 200 male respondents who have had sex in the last 3 months (Table E1), with the survey-specific response rates reported in Table E2. Information on the respondent’s characteristics, i.e., place of residence, household wealth and years of schooling, were extracted. The years of schooling variable was dichotomized as less than 6 years and 6 or more years.

The survey- and sex-specific proportions of respondents reporting the two risky behaviors (having two or more partners vs one partner and having “condomless sex” with no condom use at last sexual intercourse vs using a condom) are presented in the tables. Box and whisker plots are used to present the disaggregated proportions by the socioeconomic characteristics. The median of survey percentages of the two binary outcomes with 95% confidence intervals (CIs) by the background characteristics were computed. Exact 95% CIs for the median values were calculated from the binomial distribution. Country- and sex-specific logistic regression models for each of the two binary outcomes were used to estimate their associations with each socioeconomic characteristic, adjusting for age and socioeconomic characteristics. The adjusted odds ratios (ORs) were estimated using variance-component (random intercept) multilevel logistic regression on the pooled data to account for observed and unobserved within and between countries heterogeneity. The stratified multistage sampling design and the survey-normalized weights were taken into account in the analyses using the survey commands in Stata 15.0 (StataCorp, College Station, Texas, USA).

## Results

The total number of single, never-married adolescents aged 15–19 years who had ever had sex at the time of the survey (i.e., *the analysis sample*) ranged from 775 females in Mozambique to 1719 females in Sierra Leone, and the corresponding number of male adolescents ranged from 353 in Liberia to 1226 in Zambia (Table E1). The proportion of female adolescents who had ever had sex was the highest in Liberia (64.3%) and the lowest in Kenya (27.5%), and the corresponding proportion of male adolescents was the highest in Gabon (70.4%) and the lowest in Cameroon (28.5%). The numbers of young female and male who had sex in the last 12 and the last 3 months, and who were thus eligible for the measurement of the two behavioral outcomes, are also provided in Table E1.

Table 1 presents the prevalence and 95% confidence intervals of risky sexual behaviours for young female and male. The percentages of young female who reported having two or more partners in the last 12 months ranged from 4.3% in Angola to 15.7% in Gabon, and the corresponding percentages for the young male ranged from 11.1% in Liberia to 37.6% in Cote d’Ivoire. The percentage of young male who reported having multiple partners was much higher than that of young female (up to 4-fold in some countries) in all countries except Liberia. Condomless sex at last sexual intercourse among adolescents who had sex in the last 3 months was consistently less common among male adolescents than female adolescents, except in Mozambique. The proportions of adolescents who engaged in condomless sex ranged from 32.6% in Gabon to 93.2% in Sierra Leone among female adolescents and from 19.8% in Gabon to 84.5% in Sierra Leone among male adolescent.

**Table 1:** Prevalence and 95% confidence intervals (CIs) of risky sexual behaviors among respondents by sex

Country	Year	Multiple Sexual Partners (2+)		Condomless Sex	
		Female	Male	Female	Male
Angola	2015–16	4.3 (2.6–6.9)	18.3 (14.7–22.6)	65.1 (59.8–70.0)	56.6 (50.2–62.7)
Benin	2017–18	10.3 (8.0–13.0)	16.2 (12.1–21.3)	72.7 (68.5–76.5)	72.0 (65.4–77.7)
Cameroon	2018	13.3 (10.7–16.4)	28.8 (23.6–34.7)	42.0 (36.9–47.2)	31.5 (25.7–38.0)
Congo	2011–12	7.9 (5.7–10.9)	34.4 (28.8–40.5)	56.3 (50.0–62.4)	44.3 (38.1–50.7)
Cote d’Ivoire	2011–12	11.1 (8.5–14.3)	37.6 (30.3–45.5)	55.3 (50.2–60.3)	36.2 (28.3–44.9)
Democratic Republic of the Congo	2013–14	10.6 (8.1–13.8)	23.3 (19.0–28.3)	76.2 (70.8–80.8)	75.3 (66.6–82.3)
Gabon	2012	15.7 (11.7–20.7)	25.5 (20.6–31.1)	32.6 (27.2–38.5)	19.8 (14.4–26.6)
Kenya	2014	4.4 (2.5–7.7)	15.0 (11.6–19.2)	38.2 (30.0–47.1)	34.9 (28.1–42.4)
Liberia	2013	15.0 (11.3–19.6)	11.1 (7.0–17.2)	75.4 (69.8–80.2)	61.1 (50.5–70.8)
Malawi	2015–16	4.4 (2.9–6.6)	17.6 (14.2–21.6)	38.7 (33.4–44.2)	32.6 (27.4–38.4)
Mozambique	2011	8.4 (6.4–11.0)	28.6 (24.1–33.6)	55.1 (49.2–60.8)	59.3 (53.5–64.9)
Sierra Leone	2013	11.3 (9.4–13.5)	20.4 (16.7–24.7)	93.2 (91.3–94.8)	84.5 (80.0–88.2)
Uganda	2016	6.8 (4.9–9.3)	22.7 (17.8–28.5)	52.2 (45.9–58.5)	45.1 (36.8–53.7)
Zambia	2018	4.3 (2.8–6.6)	18.1 (15.2–21.5)	69.7 (64.0–74.8)	56.9 (52.6–61.0)

Table 2 shows the median percentages of the two risky sexual behavior indicators with the 95% CIs by place of residence, household wealth, and year of schooling for young female and male. In addition, the between-country variation in these two indicators by sex and socioeconomic characteristics are depicted in Figures E1-E5. In general, the differences in multiple partners and condomless sex between socioeconomic groups were statistically insignificant for females and males. There is no appreciable difference in the number of reports of multiple partners by place of residence; however, on average, reporting condomless sex (i.e. no condom use at most recent sexual intercourse) was 14 and 20 percentage points lower in urban female and male adolescents, respectively, compared to rural adolescents. Adolescents living in wealthy households reported more condom use at most recent sexual intercourse and males reported more multiple partnerships than those who were living in poorer households. Female adolescent with fewer than 6+ years of schooling reported having more multiple partners and engaging in condomless sex than their female educated counterparts. However, a higher percentage of males with more years of schooling reported multiple partners and a lower percentage reported condomless sex.

**Table 2:** Median percentages and 95% confidence intervals (CIs) for risky sexual behaviors by socioeconomic indicators and sex

Variable	Multiple Sexual Partners (2+)		Unprotected Sex	
	Female	Male	Female	Male
**Residence**				
Rural	10.3 (4.6–13.2)	18.6 (14.7–25.6)	68.1 (46.3–79.8)	55.6 (41.1–80.0)
Urban	9.4 (4.9–11.8)	22.8 (18.8–27.8)	51.6 (35.0–68.7)	42.3 (25.3–51.0)
**Wealth**				
Lowest	10.6 (4.4–12.4)	19.6 (14.8–28.8)	72.5 (51.4–86.8)	61.1 (46.3–81.9)
Middle	8.9 (4.8–12.8)	20.9 (16.2–28.1)	54.9 (43.6–70.0)	47.4 (36.1–63.6)
Highest	9.3 (5.4–12.1)	21.8 (17.2–27.4)	46.9 (31.3–67.0)	42.4 (23.4–47.9)
**Years of schooling**				
0–5	10.7 (7.6–15.1)	20.4 (13.0–30.0)	78.9 (60.5–85.5)	64.8 (54.9–81.7)
6+	9.3 (4.1–12.0)	21.3 (17.1–27.2)	49.9 (41.0–67.3)	45.7 (27.8–55.3)

The adjusted association between the two risky sexual behaviors and the three determinants are summarized by sex in Table 3. The survey- and sex-specific age-adjusted odds ratios (aORs) are illustrated using forest plots (Figures E4-E6) and there is considerable between country variability. The adjusted results in Table 3 show that condomless sex was strongly associated with place of residence, household wealth and years of schooling for both female and male adolescents while among male adolescents only, multiple partnerships was significantly associated with place of residence. The odds of adolescents who resided in urban settings, lived in affluent households and had 6+ year of schooling having condomless sex at last sexual intercourse were significantly lower than those of their rural poor and less educated counterparts. The odds of male adolescents who resided in urban settings having multiple partners were higher than their rural poor counterparts.

**Table 3:** Adjusted ORs^a^ and 95% confidence intervals (CIs) from multilevel random intercept models of the effect of socioeconomic indicators on risky sexual behaviors by sex

Covariate	Multiple Sexual Partners (2+)		Condomless Sex	
	Female	Male	Female	Male
**Residence**				
Rural	1	1	1	1
Urban	1.06 (0.89–1.26)	1.21 (1.04–1.41)	0.73 (0.64–0.84)	0.55 (0.46–0.65)
**Wealth**				
Lowest	1	1	1	1
Middle	1.04 (0.88–1.24)	1.02 (0.88–1.19)	0.59 (0.51–0.68)	0.69 (0.59–0.81)
Highest	0.93 (0.75–1.16)	1.04 (0.87–1.24)	0.44 (0.37–0.53)	0.52 (0.42–0.63)
**Years of schooling**				
0–5	1	1	1	1
6+	0.89 (0.76–1.05)	0.92 (0.79–1.07)	0.49 (0.43–0.56)	0.50 (0.43–0.58)
**Random effect**				
Survey	0.46 (0.30–0.70)	0.36 (0.23–0.54)	0.94 (0.64–1.37)	0.95 (0.65–1.40)
Cluster	0.69 (0.56–0.84)	0.62 (0.50–0.76)	0.56 (0.45–0.69)	0.67 (0.54–0.83)

^a^ ORs were adjusted for age and socioeconomic characteristics

## Discussion

This manuscript documented the extent and magnitude of variation in number of sexual partners and condom use by socioeconomic status among single, never-married adolescents aged 15–19 years who report having ever had sex in 14 sub-Saharan Africa countries. Female and male adolescents living in urban settings, those who had more than 6 years of schooling, and those who were wealthier were significantly more likely to use condoms at last sexual intercourse. Also, urban males had significantly higher odds for multiple sexual partners compared to rural males**.** Conversely, none of the three socioeconomic characteristics examined showed any significant association with having multiple sexual partners among women.

The HIV prevalence in sub-Saharan Africa is very high, and HIV is transmitted mainly through heterosexual acts [18]. More than elsewhere, young people in sub-Saharan Africa remain the most threatened, accounting for half of all new HIV infections [9]. Hence, focusing on young people’s sexual behavior, such as having multiple sexual partnerships, which lies at the root of the generalized epidemic of HIV [19], is crucial to understanding sexual practices and norms that affect behavior and health in adult life [20].

Aside HIV/STIs, unintended pregnancies are another import negative outcome associated with risky sexual behaviour that may have particularly strong impact not only on young women’s health but also their future lives driving them to even greater poverty and social exclusion.

The clear difference in the number of sexual partners between young female and male, with the latter reporting more than one partner in almost all countries under study, is possibly rooted in gender norms and beliefs [21]. Evidence from Nigeria and South Africa suggests that adolescent males who have many sexual partners are accorded higher social standings among their peers [ 22]. Conversely, monogamous males are often perceived in sub-Saharan Africa as lacking virility [23]. On the other hand, female engaging in multiple sexual relationships are often despised and labeled with insulting terms such as “loose” [24].

The sexual double standard, first proposed by Reis [25] in the late 1950s and recently confirmed and well described by Moreau et al. [26], as a set of rules holding young boys and girls to different standards with regards to premarital sexual interactions, is common across cultures and has profound implications especially for girls’ health and well-being by restraining their autonomy while exposing them to greater risks [26].

Also, important to note how risky behaviors among young men in urban areas have been reported in previous manuscripts and this may be referred to higher chances of experimenting with sex when compared with adolescents living in rural areas [12].

The findings showing a high proportion of condom usage at last sexual intercourse among female in countries such as Kenya and Gabon are in line with the findings reported in a previous article [27]. Regional variations might stem from differences in education approaches between countries: some might be influenced by South Africa’s policies, which allow young adults to engage in sexual activity without incurring criminal sanctions [28]. Conversely, very low usage of condoms is evidenced in West African countries as well as in the Democratic Republic of the Congo, where young adults are likely to express themselves sexually [27].

The association between more years of schooling and condom use has been reported by other studies [15]; however, it is unclear whether this association is driven by potential exposure to sexual education or other determinants influencing risky behaviors [29]. In this regard, previous evidence has shown that sex education in schools is linked to positive effects on the consistent and correct use of condoms and that there is a need for out-of-school programs aimed at providing relevant instructions before adolescent become sexually active [10]. In many African settings, the ability of poor female to negotiate the use of condoms may be undermined if they have received gifts or money [30]. This is also the case when adults take advantage of unsuspecting young adolescent female by providing basic needs and engaging in sexual relationships with them [31]. The socioeconomic situation may also influence attitudes toward risky behaviors, such as binge drinking [32], which in turn is associated with sexual risky behaviors [33], especially in adolescent males [34]. In this regard, the influence of wealth varies from country to country and may reflect different context-specific norms [35]. As reported by other studies, residential area is another key factor in the observed differences in sexual behavior, such as condom use, with the socialization process and exposure to mass media informing sexual practices and sexual health choices [11].

Our findings on higher reporting of multiple partnerships in urban male when compared to rural counterparts is in line with previous reports [14]. A previous DHS-based review found a higher proportion of sexually active adolescents who reported multiple partners in urban areas than in rural areas especially among males [15]; this difference was significant only for females in 4/11 countries [15]. Again, in only four of eleven countries, better educated girls were more likely to report having multiple partners than those with fewer years of schooling [15]. On the other hand, there were no consistent patterns among male adolescents by education level [15].

Although the DHS are considered to be good quality because of their characteristics such as their standardized procedures, some limitations are evident. We may have missed young people living on the street, enrolled in boarding schools and working in mines or brothels, which is a problem for all household surveys. Moreover, as in the analysis of self-reported behavior on sensitive topics, social desirability may have distorted the validity of the reports [36]. Social desirability bias is particularly problematic, as respondents may deliberately answer questions inaccurately, either by underreporting stigmatized activities or by overreporting normative activities if their actual behavior would be considered socially unacceptable [37]. Overall, young people, especially females, frequently underreport sexual practices, while males sometimes overreport sexual practices [38]. In our analysis, we included data from countries from different parts of Africa and different points in time across 10 years, thus possibly reflecting important differences in attitudes. In nationally representative household surveys, the commonly used indicator of condom use for adolescents is the proportion of those using a condom at last sex intercourse; considering that the risk of acquiring HIV is reduced by the consistent use of condoms [39], our indicator is a surrogate measure of consistency [15]. Finally, the wealth index developed by DHS as a proxy for household affluence has its limitations.

The present study has drawn attention to the structural factors that could help to predict adolescent sexual risk behaviors. In that sense, the findings confirm our hypothesis that education plays a crucial role in facilitating the use of condoms among male and female adolescents. The findings also confirm our hypothesis that the use of condoms is more common in urban settings, likely due to better access to condoms, and among wealthy females, due to their abilities to negotiate condom use. However, an impact of these factors on having multiple sexual partners was observed only for male adolescents with those living in urban settings having higher odds for multiple sexual partners compared to those in rural areas.

Our study reinforces the notion that interventions need to be targeted based on characteristics such as sex, age, literacy and residence. The practice of protected sex, abstinence and partner reduction is vital in preventing sexually transmitted infections, including HIV, as are monitoring intervention programs to control the spread of HIV/STIs among adolescents. While several countries in Africa currently have education policies or strategies making basic education compulsory and free, most countries do not cover the associated costs of schooling, which acts as a barrier, particularly for girls [40]. Many countries also have policies to include sexual health and HIV in educational curricula; however, the implementation of these policies is often uneven [38]. Moreover, there is often a lack of clarity in national policies about the age at which girls are legally allowed to access contraceptives, which may represent an important barrier in countries such as Sierra Leone, where the age of consent is high [40]. School-based prevention intervention programs [41] would be beneficial in delaying sexual debut and increasing condom use. Effective strategies and programming for adolescent sexual health are available; nevertheless, increased effort is needed to enforce the existing policies and scale-up successful initiatives.

## Supplementary Information

**Additional file 1.**

## Data Availability

The datasets analyzed during the current study are publicly available at www.measuredhs.com.
