# Supplementary material for: Socioeconomic variations in risky sexual behavior among adolescents in 14 sub-Saharan Africa countries who report ever having had sex
Source: Int J Equity Health. 2021 Jan 6;20:11. doi: 10.1186/s12939-020-01352-8 (PMC7789383; doi:10.1186/s12939-020-01352-8)
Supplement: Supplementary file 1 — Additional file 1. [file 12939_2020_1352_MOESM1_ESM.docx]

**Table E1.** Numbers of all respondents; all single, never-married adolescent respondents; and, among the single adolescents, those who ever had sex and those who had sex in the last 12 and 3 months by sex.

| **Country** | **Year** | **Female** | | | | |  | **Male** | | | | |
| --- | --- | --- | --- | --- | --- | --- | --- | --- | --- | --- | --- | --- |
|  |  | **Respondents (aged 15-49)** | **Single never-married adolescents (aged 15-19)** | | | |  | **Respondents (aged 15-49)** | **Single never-married adolescents (aged 15-19)** | | | |
|  |  |  | **Total** | **Ever had sex** | **Had sex in the last 12** | **Had sex in the last 3 months** |  |  | **Total** | **Ever had sex** | **Had sex in the last 12** | **Had sex in the last 3 months** |
| Angola | 2015-16 | 14,379 | 2,761 | 1,427 | 1,029 | 775 |  | 5,684 | 1,428 | 939 | 710 | 569 |
| Benin | 2017-18 | 15,928 | 2,722 | 977 | 820 | 594 |  | 7,595 | 1,538 | 440 | 331 | 240 |
| Cameroon | 2018 | 14,677 | 2,648 | 801 | 645 | 482 |  | 6,978 | 1,504 | 429 | 361 | 227 |
| Congo | 2011-12 | 10,819 | 1,671 | 877 | 760 | 616 |  | 5,145 | 969 | 606 | 551 | 451 |
| Cote d'Ivoire | 2011-12 | 10,060 | 1,586 | 859 | 690 | 557 |  | 5,135 | 858 | 359 | 301 | 238 |
| Democratic Republic of the Congo | 2013-14 | 18,827 | 3,083 | 1,125 | 866 | 642 |  | 8,656 | 1,707 | 777 | 618 | 480 |
| Gabon | 2012 | 8,422 | 1,516 | 885 | 741 | 577 |  | 5,654 | 997 | 702 | 574 | 410 |
| Kenya | 2014 | 31,079 | 5,052 | 1,388 | 360 | 208 |  | 12,819 | 2,522 | 1,010 | 625 | 388 |
| Liberia | 2013 | 9,239 | 1,752 | 1,127 | 955 | 835 |  | 4,118 | 877 | 353 | 326 | 248 |
| Malawi | 2015-16 | 24,562 | 3,851 | 1,322 | 836 | 524 |  | 7,478 | 1,757 | 904 | 634 | 490 |
| Mozambique | 2011 | 13,745 | 1,806 | 775 | 621 | 513 |  | 4,035 | 805 | 479 | 462 | 372 |
| Sierra Leone | 2013 | 16,658 | 3,114 | 1,719 | 1,490 | 1,311 |  | 7,262 | 1,460 | 639 | 573 | 458 |
| Uganda | 2016 | 18,506 | 3,292 | 975 | 655 | 397 |  | 5,336 | 1,256 | 520 | 346 | 219 |
| Zambia | 2018 | 13,683 | 2,531 | 1,015 | 612 | 415 |  | 12,132 | 2,749 | 1,226 | 880 | 647 |

**Table E2.** Household and female and male response rates

| **Country** | **Year** | **Household reponse rate** | **Eligible women's response rate** | **Eligible men's reponse rate** |
| --- | --- | --- | --- | --- |
| Angola | 2015-16 | 99.4 | 96 | 94.2 |
| Benin | 2017-18 | 99 | 98.1 | 97.6 |
| Cameroon | 2018 | 99.4 | 98.1 | 97.5 |
| Congo | 2011-12 | 99.8 | 98 | 96.8 |
| Cote d'Ivoire | 2011-12 | 98.1 | 92.7 | 90.5 |
| Democratic Republic of the Congo | 2013-14 | 99.9 | 98.6 | 97.4 |
| Gabon | 2012 | 99.3 | 98.2 | 96.2 |
| Kenya | 2014 | 99 | 96.6 | 90.2 |
| Liberia | 2013 | 99.4 | 97.6 | 95.4 |
| Malawi | 2015-16 | 99.2 | 97.7 | 94.6 |
| Mozambique | 2011 | 99.8 | 99.1 | 97.7 |
| Sierra Leone | 2013 | 99.3 | 97.2 | 96.4 |
| Uganda | 2016 | 98.2 | 97 | 94 |
| Zambia | 2018 | 99.1 | 96.4 | 91.6 |

ICF, 2015. The DHS Program STATcompiler. Funded by USAID. http://www.statcompiler.com. November 16 2020

**Fig. E1a.** Proportion of respondents with multiple sexual partners (2+) by place of residence.

**Fig. E1b.** Proportion of respondents with multiple sexual partners (2+) by household wealth.

**Fig. E1c.** Proportion of respondents with multiple sexual partners (2+) by year of schooling.

**Fig. E2a.** Proportion of respondents who had unprotected sex at last sexual intercourse by place of residence.

**Fig. E2b.** Proportion of respondents who had unprotected sex at last sexual intercourse by household wealth.

**Fig. E2c.** Proportion of respondents who had unprotected sex at last sexual intercourse by year of schooling.

**Fig. E3.** Adjusted odds ratios and 95% confidence intervals for the effect of the socioeconomic indicators on having multiple sexual partners (2+).

**Fig. E4.** Adjusted odds ratios and 95% confidence intervals for the effect of the socioeconomic indicators on having unprotected sex.

**Fig. E5.** Odds ratios and 95% confidence intervals for the effect of residence/household wealth and multiple sexual partners (2+).

**Fig. E6.** Odds ratios and 95% confidence intervals for the effect of residence/household wealth and condom use.
